# Temperature and Malaria Trends in Highland East Africa

**DOI:** 10.1371/journal.pone.0024524

**Published:** 2011-09-15

**Authors:** David I. Stern, Peter W. Gething, Caroline W. Kabaria, William H. Temperley, Abdisalan M. Noor, Emelda A. Okiro, G. Dennis Shanks, Robert W. Snow, Simon I. Hay

**Affiliations:** 1 Crawford School of Economics and Government, Australian National University, Canberra, Australian Capital Territory, Australia; 2 Spatial Ecology and Epidemiology Group, Department of Zoology, University of Oxford, Oxford, United Kingdom; 3 Malaria Public Health and Epidemiology Group, Centre for Geographic Medicine, Kenya Medical Research Institute – University of Oxford - Wellcome Trust Collaborative Programme, Kenyatta National Hospital Grounds, Nairobi, Kenya; 4 Australian Army Malaria Institute, Enoggera, Queensland, Australia; University of Liverpool, United Kingdom

## Abstract

There has been considerable debate on the existence of trends in climate in the highlands of East Africa and hypotheses about their potential effect on the trends in malaria in the region. We apply a new robust trend test to mean temperature time series data from three editions of the University of East Anglia's Climatic Research Unit database (CRU TS) for several relevant locations. We find significant trends in the data extracted from newer editions of the database but not in the older version for periods ending in 1996. The trends in the newer data are even more significant when post-1996 data are added to the samples. We also test for trends in the data from the Kericho meteorological station prepared by Omumbo *et al*. We find no significant trend in the 1979-1995 period but a highly significant trend in the full 1979-2009 sample. However, although the malaria cases observed at Kericho, Kenya rose during a period of resurgent epidemics (1994-2002) they have since returned to a low level. A large assembly of parasite rate surveys from the region, stratified by altitude, show that this decrease in malaria prevalence is not limited to Kericho.

## Introduction

Controversy over the cause of malaria resurgences reported in the late 1990s in some areas of highland East Africa continues despite reports of an overall global reduction in prevalence of the disease [1], marked declines across many well studied communities in East Africa [2], and at the majority of seventeen non-lakeside hospitals across Kenya [3]. Using the database developed by the Climate Research Unit at the University of East Anglia (CRU), Hay *et al*. [4] tested for trends in the time series of a range of climate variables from various locations in East Africa where increases in the prevalence of malaria had been indicated. They found no evidence of a significant increase in temperature at any of the locations and some evidence of an increase in rainfall at some locations. The inference was made that, as there was no significant warming in these East African locations, global warming was unlikely to be responsible for the increases in malaria admissions seen at facilities in these locations. Rather, a host of other plausible causes could have been responsible, among which the rise of anti-malarial drug resistance was viewed as most plausible [5]. This has sparked considerable debate.

Chaves and Koenraadt [6] claim (incorrectly) that, because only one test - the Q test [7] - was used to test for serial correlation in the residuals, the procedures used by Hay *et al*. [4] were not robust. They applied three different time series methods to CRU data interpolated for Kericho and find increases in temperature in the period from 1966 to 1996. We will show in this paper that the differences between their results and our earlier work are due to differences in data rather than differences in methods. Pascual *et al*. [8] applied two time series methods to data for the four locations analyzed by Hay *et al*. [4] for the period 1950 to 2002, finding positive and significant trend components. They suggest that the differing results of Hay *et al*. [4] may stem from the differencing used in the Dickey-Fuller regression or from the method of treating seasonality with dummy variables. As the Dickey-Fuller procedure does not actually difference the data, this cannot explain the differences in findings.

By contrast, Omumbo *et al*. [9] argue that this debate is due to the inappropriate use of regionally interpolated data in place of actual observations from individual meteorological stations. Omumbo *et al*. show that the number of stations used to construct the CRU temperature database has fallen very strongly in the Kericho, Kenya region since the mid 1990s, calling into question the usefulness of the database for recent years. In collaboration with the Kenyan meteorological service, Omumbo *et al*. prepared quality-controlled series for temperature and rainfall at Kericho that takes into account the shift in the location of the weather station in 1986. For the period 1979-2009, they find that mean temperature increased by 0.21 K per decade, which they claim is significant at the 1% level.

Given the different, but formally untested, arguments made against the Hay *et al*. [4] results and the different methodologies used, it is still unclear why there is a difference between the results of these more recent studies and our earlier work. Because of the importance of this debate [10], we apply a uniform methodology to test for trends in the various datasets used to date and time periods previously considered in the literature to determine whether differences in data or in methods are responsible for the variant findings. We find that it is differences in data examined, not methods, that is responsible.

We apply a relatively new test for temporal trends that is robust to autocorrelation of unknown form including a possible random walk in the regression residuals [11], [12]. We test the original monthly time series from the Climate Research Unit Time Series (CRU TS) 1.0 data set [13] used by Hay *et al*. [4] for four locations in highland East Africa, as well as the more recently published CRU TS 2.1 data [14] used by Chaves and Koenraadt [6] and Pascual *et al*. [8], and the newest data set, CRU TS 3.0. For these newer datasets we also test for trends in the regional average temperature across East Africa. Additionally, we test for trends in the data from the Kericho meteorological station prepared by Omumbo *et al*. [9].

We also apply the new trend test to various sub-samples of an updated time series of malaria cases at a tea estate hospital in Kericho, Kenya, that has formed the centerpiece of much of the debate regarding climate change and malaria in the highlands of East Africa [6], [9], [15]. Finally, through the data collection initiatives of the Malaria Atlas Project (MAP, http://www.map.ox.ac.uk) [16] we are able to present information on more than 5000 geo-positioned, post-1985, malaria prevalence surveys that allow insights into the changing endemicity of malaria in several East African countries during the last 25 years and if these show any difference by altitude.

## Methods

### Time Series Data and Samples

**Figure 1:**
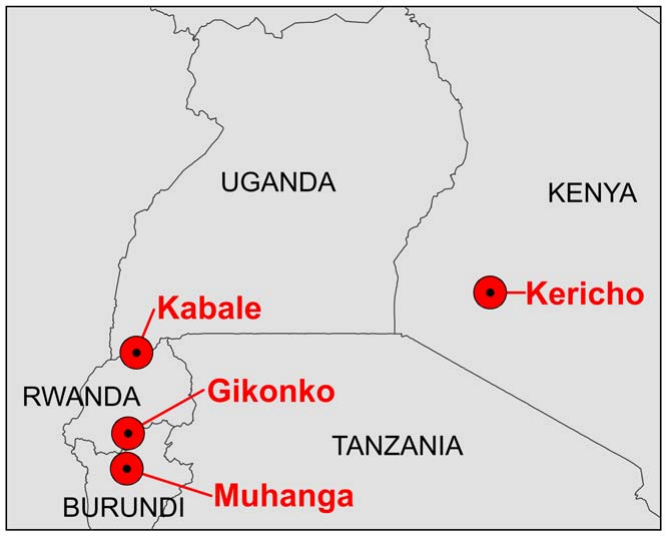
Location map. Sites at which monthly temperature data were analyzed are shown in red. Countries for which *P. falciparum* parasite rate data were analyzed are labeled. The map extent exactly matches the area defined by Patz *et al*. [19] across which CRU TS 2.1 and 3.0 data were tested: 4°S, 4°N, 28°E, 38°E.

We test mean monthly temperature data for the four locations (Kericho, Kenya; Kabale, Uganda; Gikongoro, Rwanda; and Muhanga, Burundi) examined by Hay *et al*. (2002a) (Figure 1). We also test the CRU TS 2.1 and 3.0 data for an area of East Africa defined by Patz *et al* [17] (Figure 1) to investigate whether similar results are also manifest across the region. Though much debate has focused on Kericho in Kenya, broader trends across highland East Africa should really be of interest in the debate about the role of climate change in malaria incidence. All sample sizes can be computed from the number of months in the period considered.

Since the data and periods examined in the literature have become somewhat numerous, a series of tests are required to explore the reasons for differences between results obtained in the various studies. The data samples considered are: (A) the actual data (CRU TS 1.0) used by Hay *et al*. [4] for 1970:1-1995:12; (B) the newer dataset (CRU TS 2.1) used by Chaves and Koenraadt [6] for 1966:1-1996:12, which provides a direct test of Chaves and Koenraadt's claims using our methods; (C) these same data (CRU TS 2.1) for 1950:1-2002:12 to provide a direct test of the claims of Pascual *et al*. [8] using our methods; (D) the CRU TS 2.1 data for the period 1970:1-1995:12 as a direct test of the effect of the change in data source on Hay *et al*.'s [4] results; (E) the new dataset (CRU TS 3.0) for 1970:1-1995:12 to test if there are any differences in moving from CRU TS 2.1 to CRU TS 3.0; (F) the CRU TS 3.0 series for 1966:1-1995:12 in order to test if adding earlier years influenced the results; (G) the CRU TS 3.0 for 1966:1-2006:6 to test whether the trend in temperature persists or accelerates after 1995; (H) Omumbo *et al*.'s data for 1979:1-1995:12; and (I) Omumbo *et al*.'s data for 1979:1-2009:12.

**Figure 2:**
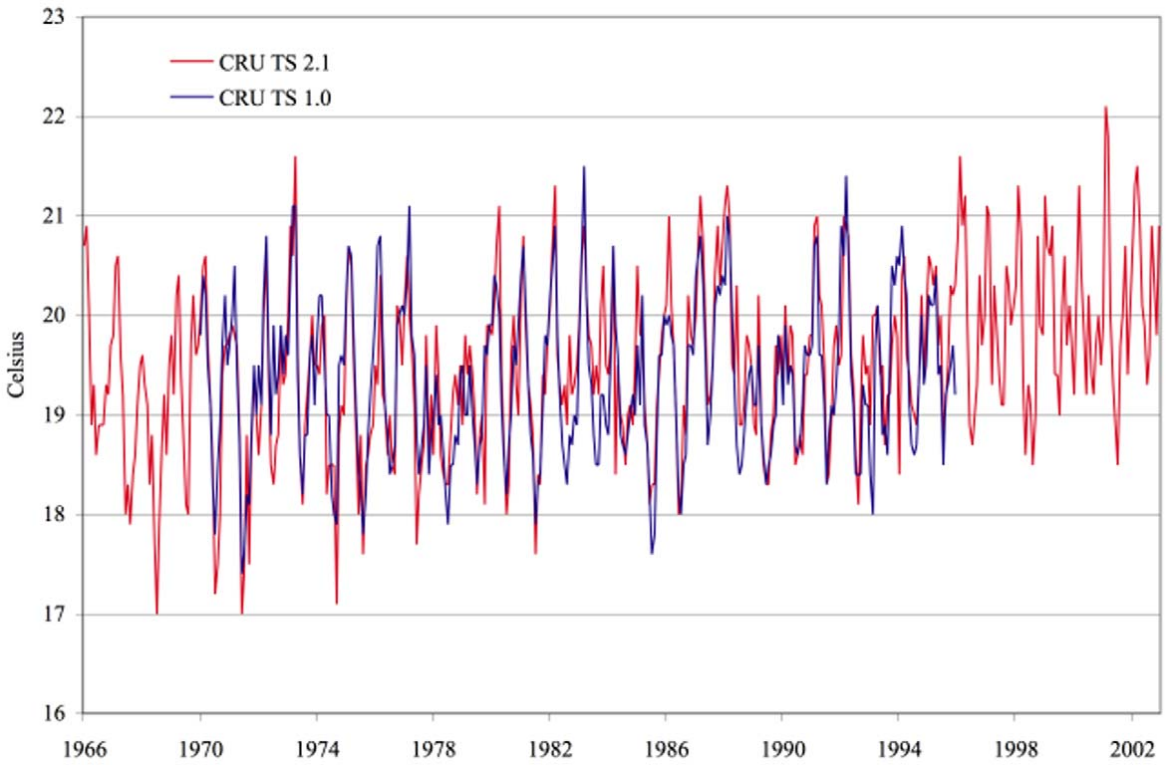
Temperature series for Kericho: CRU TS 1.0 vs. CRU TS 2.1.

**Figure 3:**
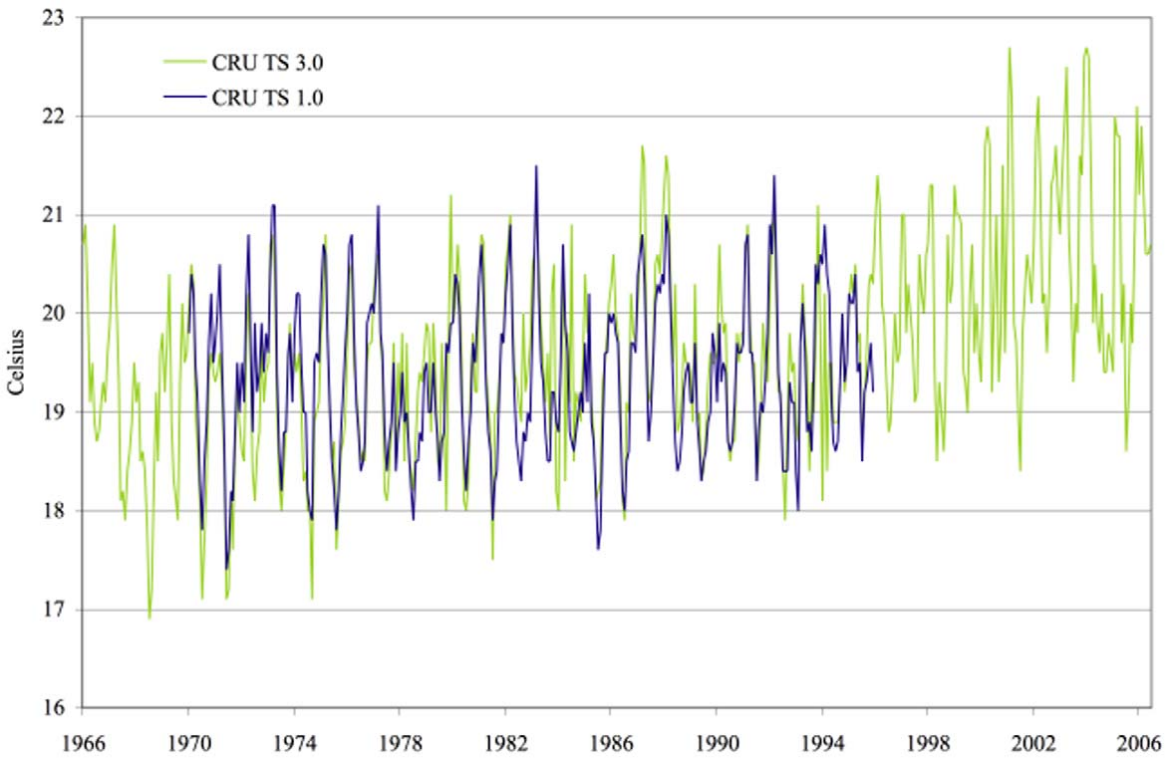
Temperature series for Kericho: CRU TS 1.0 vs. CRU TS 3.0.

The CRU TS 2.1 and TS 3.0 data are similar to the data we used previously for the period 1970-1995 but there are differences. In particular, the new series trend more. Figure 2 compares CRU TS 2.1 to CRU TS 1.0 and Figure 3 compares CRU TS 3.0 to CRU TS 1.0 for the Kericho location. During the 1970s, the cold season temperatures tend to be lower in the new series, while in the 1980s the warm season temperatures tend to be higher. An increase in temperature after 1995 is also apparent from both figures.

**Figure 4:**
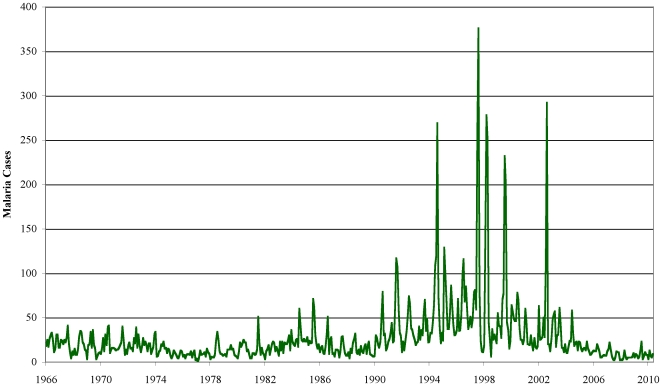
Monthly malaria cases at Kericho Unilever Tea Kenya Ltd Hospital.

We also test for trends in a series of the monthly number of malaria cases from the Unilever Tea Kenya Ltd (UTKL) tea estate hospital in Kericho using the robust trend tests (Figure 4). We test this data for the following periods: (a) 1966:1-1995:1, the sample period covered by Hay *et al*. [4] who tested for trends in the same series using the Dickey Fuller regression and suite of test statistics; (b) 1966:1-2006:6 to correspond to the temperature sample from test “G” above and (c) 1966:1-2010:5, a period that includes the most recent malaria data.

### Community Parasite Rate Surveys

As an additional indication of wider temporal patterns in malaria endemicity across East Africa, data for the region were extracted from the MAP database of community parasite rate surveys spanning the years 1985 to 2010 [18]. A total of 2,443 such surveys were available for Kenya, 2,084 for Tanzania, and a total of 721 for Burundi, Rwanda, and Uganda which were pooled for analysis. The observed parasite rate in each survey was first standardized to the epidemiologically informative two-up-to-ten year old age group using a previously described age-adjustment algorithm [19], but no adjustments were made for sample size or whether the surveys refer to urban or rural areas. In each of the three groupings, temporal changes in observed parasite prevalence were summarized graphically by plotting the median and inter-quartile range of surveyed values in each year, accompanied by a moving-average line generated with the LOWESS smoother [20]. Importantly, these summaries were calculated separately for low (<1500 m) and high (>1500 m) altitude areas.

### Time Series Methods

The *t_DAN_* [11] or Dan-J test [12] is based on a modified t-test on the slope parameter of the simple linear trend regression model:
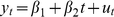
(1)where *t* is a linear time trend, *u*, is a stochastic process that may or may not be stationary and 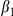 and 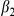 are regression parameters to be estimated. Then the trend test statistic is given by:
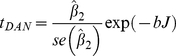
(2)where 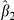 is the estimate of the slope parameter in (1) and 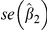 its standard error. *b* is a parameter derived by Bunzel and Vogelsang [12] and:
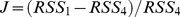
(3)where *RSS_1_* is the sum of squared residuals from (1), and *RSS_4_* is the sum of squared residuals from the following regression:
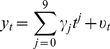
(4)


The standard error of the slope parameter, 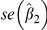, is computed as follows:
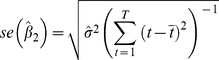
(5)with 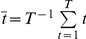, *T* is the sample size, and:
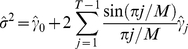
(6)where 
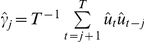
 is a function of the estimated residuals 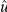, and *M* =  max(0.02T,2).

The recommended value of b and the critical values of *t_DAN_* for a two-tailed test are as follows: *b* = 2.466, *t_DAN_* = 2.462 at 1%; *b* = 1.795, *t_DAN_* = 2.052 at 2.5%; *b* = 1.322, *t_DAN_* = 1.710 at 5% and *b* = 0.965, *t_DAN_* = 1.329 at the 10% significance level. Further values can be derived from the formulae in [12]. *J* can also be used in a left-tailed test of the null hypothesis that the errors in (1) contain a unit root autoregressive process or random walk. The null hypothesis is rejected for **small** values of the statistic. The critical values are 0.488 at 1%, 0.678 at 2.5%, and 0.908 at the 5% significance levels. We also carried out the *t_DAN_* test on deseasonalized data constructed by removing monthly means from the data.

## Results

Table 1 presents the results of the robust trend tests for the unadjusted temperature series. For the CRU TS 1.0 data used by Hay *et al*. [4], the actual temperature changes estimated by the trend component vary from 0.21 K for Kericho to 0.27 K for Gikongoro and Muhanga. None of these changes are statistically significant at the 5% level as determined by the *t_DAN_* statistics and only Muhanga and Gikongoro have a statistically significant trend at the 10% level. By contrast, the CRU TS 2.1 data used by Chaves and Koenraadt [6] show highly significant trends for all four sites and the East Africa region as a whole for the 1966 to 1996 period that they used. The increases in temperature over the period are much higher than we found for the 1970-1995 period using the CRU TS 1.0 series. Moreover, when we restrict the CRU TS 2.1 sample to the latter narrower window the temperature increases are still higher for the four locations, though not for East Africa as a whole. The test statistics are also highly significant when we consider the CRU TS 2.1 data for the period 1950-2002 used by Pascual *et al* [8].

**Table 1:** Vogelsang Trend Tests for Unadjusted CRU Temperature Series.

			*t_DAN_* for a test of size:				
	Slope 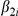	Change in temp.	1%	2.5%	5%	10%	*J*
CRU TS 1.0 1970-1995							
Gikongoro	0.0009	0.27 K	1.0292	1.1722	1.2849	**1.3770**	0.1940
Kabale	0.0008	0.24 K	0.9372	1.0566	1.1498	1.2255	0.1787
Kericho	0.0007	0.21 K	0.9854	1.0038	1.0169	1.0270	0.0276
Muhanga	0.0009	0.27 K	1.3586	1.4364	1.4939	**1.5388**	0.0830
CRU TS 2.1 1966-1996							
Gikongoro	0.0024	1.33 K	**6.6471**	**6.7271**	**6.7841**	**6.8275**	0.0124
Kabale	0.0022	1.26 K	**6.2383**	**6.3129**	**6.3661**	**6.4065**	0.0142
Kericho	0.0020	1.10 K	**4.5672**	**4.6241**	**4.6647**	**4.6955**	0.0141
Muhanga	0.0024	1.32 K	**6.9302**	**6.9798**	**7.0150**	**7.0417**	0.0084
East Africa	0.0024	1.33 K	**6.3690**	**6.3906**	**6.4058**	**6.4174**	0.0047
CRU TS 2.1 1950-2002							
Gikongoro	0.0014	0.88 K	**5.2578**	**5.5488**	**5.7635**	**5.9311**	0.0801
Kabale	0.0014	0.91 K	**5.6928**	**5.9445**	**6.1286**	**6.2713**	0.0644
Kericho	0.0009	0.58 K	**2.8030**	**2.9514**	**3.0607**	**3.1458**	0.0764
Muhanga	0.0014	0.87 K	**5.4726**	**5.6860**	**5.8415**	**5.9616**	0.0568
East Africa	0.0014	0.86 K	**5.1451**	**5.3455**	**5.4913**	**5.6041**	0.0558
CRU TS 2.1 1970-1995							
Gikongoro	0.0030	1.63 K	**7.8737**	**7.9935**	**8.0791**	**8.1442**	0.0207
Kabale	0.0027	1.51 K	**7.2907**	**7.3972**	**7.4733**	**7.5312**	0.0188
Kericho	0.0021	1.14 K	**4.4185**	**4.4565**	**4.4835**	**4.5039**	0.0127
Muhanga	0.0030	1.64 K	**8.2203**	**8.3123**	**8.3778**	**8.4276**	0.0147
East Africa	0.0024	1.33 K	**5.8016**	**5.8580**	**5.8981**	**5.9285**	0.0144
CRU TS 3.0 1970-1995							
Gikongoro	0.0011	0.38 K	2.3754	**2.4673**	**2.5341**	**2.5858**	0.0257
Kabale	0.0008	0.30 K	1.8219	1.8855	**1.9317**	**1.9673**	0.0218
Kericho	0.0024	0.87 K	**3.9763**	**4.1289**	**4.2401**	**4.3260**	0.0309
Muhanga	0.0013	0.46 K	**3.0381**	**3.1153**	**3.1709**	**3.2135**	0.0171
East Africa	0.0015	0.53 K	**3.2572**	**3.3316**	**3.3849**	**3.4258**	0.0135
CRU TS 3.0 1966-1995							
Gikongoro	0.0009	0.31 K	**2.4917**	**2.5957**	**2.6716**	**2.7304**	0.0202
Kabale	0.0007	0.25 K	1.9526	2.0271	**2.0813**	**2.1232**	0.0163
Kericho	0.0021	0.76 K	**4.4329**	**4.6279**	**4.7704**	**4.8809**	0.0414
Muhanga	0.0011	0.38 K	**3.2002**	**3.2849**	**3.3459**	**3.3928**	0.0143
East Africa	0.0016	0.56 K	**4.2935**	**4.4178**	**4.5076**	**4.5765**	0.0105
CRU TS 3.0 1966-2006							
Gikongoro	0.0029	1.41 K	**3.3042**	**4.0075**	**4.5913**	**5.0877**	0.2146
Kabale	0.0030	1.44 K	**3.1192**	**3.8203**	**4.4072**	**4.9092**	0.2361
Kericho	0.0035	1.69 K	**7.5176**	**7.9318**	**8.2374**	**8.4758**	0.0653
Muhanga	0.0029	1.38 K	**4.4023**	**4.9657**	**5.4056**	**5.7633**	0.1300
East Africa	0.0032	1.54 K	**6.6389**	**7.1417**	**7.5188**	**7.8166**	0.0842

Note: Significant t-statistics are marked in bold. *t_DAN_* test is for significance of the trend and *J* is a test for a unit root in the residuals of the trend regression. The change in temperature is the trend coefficient multiplied by the length of the sample period.

Compared to the data we used previously, the CRU TS 3.0 data also show uniformly greater temperature increases and more significant t-statistics, especially for Kericho, though the changes are smaller than in the CRU TS 2.1 data. In the 1966 to 1995 period we find a 0.76 K temperature increase at Kericho, which is significant at the 1% level. The temperature increase in Kabale is significant at the 5% level and in the other two locations and across rhe region at the 1% level. Increasing the start date to 1970 results in similar but slightly steeper slope coefficients. Trends are significant at the 5% level in all four locations but at the 1% level only in Kericho and Muhanga. For 1966 to 2006, the temperature increases are larger still, ranging from 1.38 K to 1.69 K. The increase is significant at the 1% level at all locations.

All of the *J* statistics used to test for stochastic trends in the data are highly significant at the 1% level allowing us to reject the null hypothesis of a unit root in the residuals of the trend regression. The results for deseasonalized data are almost identical to those for the unadjusted data for most of the samples (Table 2) showing that the seasonal cycle does not have a strong influence on our findings for the CRU datasets.

**Table 2:** Vogelsang Trend Tests for Deseasonalized CRU Temperature Series.

			*t_DAN_* for a test of size:				
	Slope 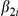	Change in temp.	1%	2.5%	5%	10%	*J*
CRU TS 1.0 1970-1995							
Gikongoro	0.0009	0.27 K	0.8787	1.0499	1.1903	1.3086	0.2653
Kabale	0.0008	0.26 K	0.8340	0.9914	1.1198	1.2276	0.2575
Kericho	0.0008	0.24 K	1.1363	1.1771	1.2068	1.2297	0.0526
Muhanga	0.0008	0.26 K	1.0309	1.1744	1.2874	**1.3798**	0.1942
CRU TS 2.1 1966-1996							
Gikongoro	0.0024	1.34 K	**6.4649**	**6.5960**	**6.6900**	**6.7618**	0.0299
Kabale	0.0023	1.29 K	**6.2620**	**6.3754**	**6.4566**	**6.5186**	0.0268
Kericho	0.0020	1.14 K	**4.2978**	**4.4968**	**4.6425**	**4.7556**	0.0674
Muhanga	0.0023	1.31 K	**6.5475**	**6.6840**	**6.7820**	**6.8569**	0.0308
East Africa	0.0024	1.37 K	**5.8627**	**6.0960**	**6.2660**	**6.3974**	0.0582
CRU TS 2.1 1950-2002							
Gikongoro	0.0014	0.89 K	**4.9150**	**5.2856**	**5.5635**	**5.7829**	0.1083
Kabale	0.0014	0.92 K	**5.2395**	**5.6091**	**5.8852**	**6.1026**	0.1016
Kericho	0.0009	0.60 K	2.0589	**2.3727**	**2.6222**	**2.8277**	0.2114
Muhanga	0.0014	0.86 K	**4.7717**	**5.1432**	**5.4223**	**5.6430**	0.1117
East Africa	0.0014	0.88 K	**3.7783**	**4.2849**	**4.6824**	**5.0066**	0.1875
CRU TS 2.1 1970-1995							
Gikongoro	0.0030	1.63 K	**7.6104**	**7.8115**	**7.9564**	**8.0676**	0.0389
Kabale	0.0028	1.54 K	**7.0721**	**7.2872**	**7.4427**	**7.5622**	0.0446
Kericho	0.0022	1.19 K	**4.6557**	**4.7620**	**4.8384**	**4.8969**	0.0336
Muhanga	0.0029	1.62 K	**7.8518**	**8.0705**	**8.2284**	**8.3496**	0.0410
East Africa	0.0025	1.38 K	**5.8082**	**6.0044**	**6.1466**	**6.2562**	0.0495
CRU TS 3.0 1970-1995							
Gikongoro	0.0011	0.38 K	2.3702	**2.5033**	**2.6015**	**2.6782**	0.0814
Kabale	0.0009	0.32 K	1.9347	2.0436	**2.1239**	**2.1867**	0.0816
Kericho	0.0025	0.90 K	**4.0753**	**4.3941**	**4.6338**	**4.8233**	0.1123
Muhanga	0.0012	0.44 K	**2.8252**	**2.9805**	**3.0950**	**3.1843**	0.0797
East Africa	0.0016	0.56 K	**3.8502**	**4.1209**	**4.3231**	**4.4822**	0.1013
CRU TS 3.0 1966-1995							
Gikongoro	0.0009	0.31 K	2.2580	**2.3746**	**2.4605**	**2.5273**	0.0751
Kabale	0.0007	0.26 K	1.8229	1.9181	**1.9881**	**2.0426**	0.0758
Kericho	0.0022	0.78 K	**3.8628**	**4.1288**	**4.3272**	**4.4832**	0.0992
Muhanga	0.0010	0.37 K	**2.6822**	**2.8150**	**2.9125**	**2.9883**	0.0720
East Africa	0.0016	0.58 K	**3.1164**	**3.2948**	**3.4266**	**3.5296**	0.0830
CRU TS 3.0 1966-2006							
Gikongoro	0.0029	1.41 K	**2.9338**	**3.6795**	**4.3164**	**4.8692**	0.3375
Kabale	0.0030	1.44 K	**2.5920**	**3.3404**	**3.9944**	**4.5715**	0.3780
Kericho	0.0035	1.69 K	**6.5238**	**7.1740**	**7.6709**	**8.0686**	0.1416
Muhanga	0.0029	1.39 K	**3.3252**	**4.0542**	**4.6623**	**5.1809**	0.2954
East Africa	0.0032	1.54 K	**4.7512**	**5.6054**	**6.2983**	**6.8774**	0.2464

Note: Significant t-statistics are marked in bold. *t_DAN_* test is for significance of the trend and *J* is a test for a unit root in the residuals of the trend regression. The change in temperature is the trend coefficient multiplied by the length of the sample period.

Table 3 presents results for Omumbo *et al*.'s mean temperature series. For the unadjusted data there is no significant trend in the 1979-1995 period but there is a highly significant trend for 1979-2009. For the deseasonalized data there is a significant trend in both periods. So here, deseasonalization has an effect on the results.

**Table 3:** *t_DAN_* Trend Tests for Omumbo *et al*. Kericho Temperature Series.

			*t_DAN_* for a test of size:				
	Slope 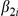	Change in temp.	1%	2.5%	5%	10%	*J_T_*
Unadjusted data 1979-1995	0.0011	0.23 K	1.2824	1.2941	1.3024	1.3087	0.0135
Unadjusted data 1979-2009	0.0018	0.68 K	**5.4096**	**5.4865**	**5.5414**	**5.5832**	0.0210
Deseasonalized data 1979-1995	0.0014	0.28 K	**3.0743**	**3.1632**	**3.2274**	**3.2767**	0.0425
Deseasonalized data 1979-2009	0.0019	0.69 K	**4.7390**	**4.9963**	**5.1860**	**5.3340**	0.0788

Note: Significant *t_DAN_* statistics marked in bold. *t_DAN_* is a test for significance of the trend and is a test for a unit root in the residuals of the trend regression. The change in temperature is the trend coefficient multiplied by the length of the sample period.

Results for the Kericho hospital malaria inpatient series are shown in Table 4. The *t_DAN_* trend test finds a significant trend in malaria cases for 1966-1995 at either the 1% or 2.5% level depending on whether the data were deseasonalized or not. The slope coefficient is positive and implies an increase in monthly incidence over the period of 29 cases per month. The maximum number of monthly cases seen in this period was 269 in August 1994 with an average of 23 per month. The major upsurge in cases came towards the end of the period. The trend coefficient for 1966-2006 is about the same as that for 1966-1995 with an implied increase over the full period of 40 cases per month but is significant at the 5% level. The average number of cases over the period was 29 per month. The maximum number of monthly cases of 376 was recorded in August 1997 soon after the end of the earlier sample. Again, the deseasonalized results are very similar. Adding the four most recent years of data, however, reduces the estimated trend coefficient to 0.0436, which is only significant at the 10% level. The overall increase in malaria cases is reduced to 23 but in the last four years only July 2009 saw that many cases and the average number of cases over the last four years has been just eight.

**Table 4:** Robust Trend Tests for Malaria Series.

			*t_DAN_* for a test of size				
Sample Period	Slope 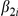	Change in Malaria Cases	1%	2.5%	5%	10%	*J*
**Unadjusted Data**							
1966:1-1995:1	0.0810	29	**2.5660**	**3.2274**	**3.7938**	**4.2862**	0.3418
1966:1-2006:6	0.0834	40	2.4009	**2.8119**	**3.1433**	**3.4190**	0.2355
1966:1-2010:5	0.0436	23	0.9523	1.2006	1.4135	**1.5989**	0.3452
**Deseasonalized Data**							
1966:1-1995:1	0.0806	29	2.0569	**2.7454**	**3.3650**	**3.9238**	0.4303
1966:1-2006:6	0.0839	41	2.2154	**2.6583**	**3.0227**	**3.3305**	0.2716
1966:1-2010:5	0.0440	24	0.8577	1.1158	1.3432	**1.5450**	0.3921

Note: Significant t-statistics are marked in bold. *t_DAN_* test is for significance of the trend and *J* is a test for a unit root in the residuals of the trend regression.

**Figure 5:**
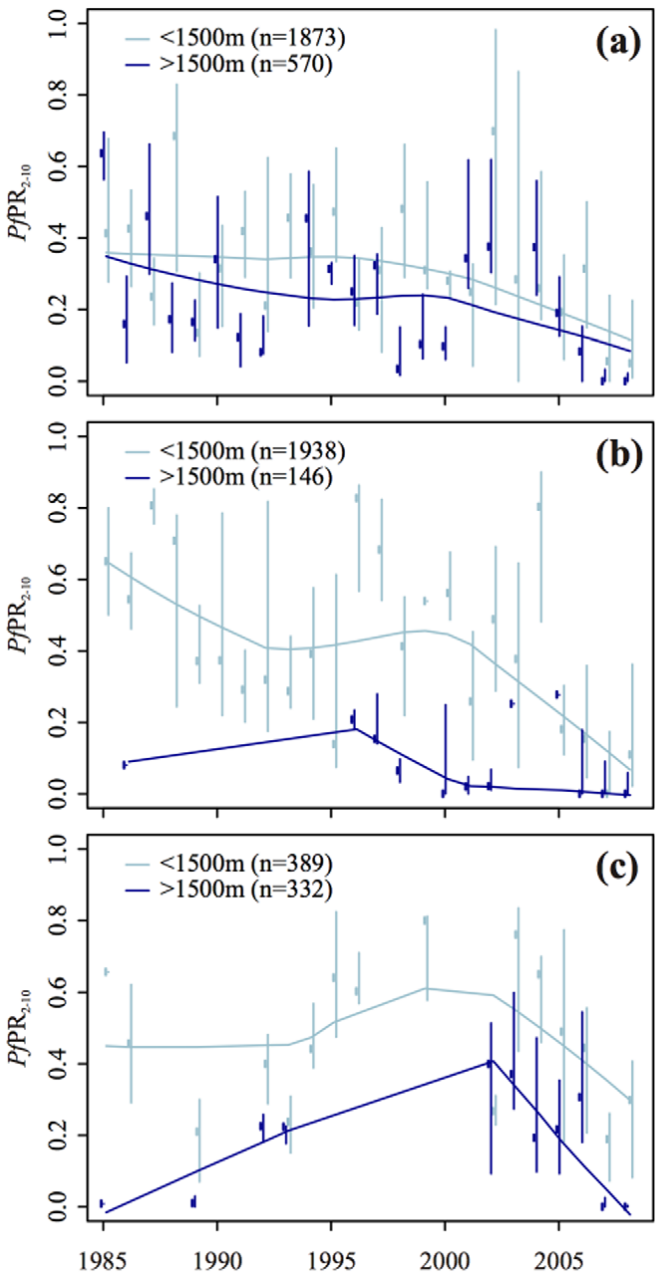
*Plasmodium falciparum* parasite rate survey data by year for five East African countries. Time series plots summarizing available community *P. falciparum* parasite rate survey data for (top) Kenya, (middle) Tanzania and (bottom) the combined region of Burundi, Rwanda, and Uganda. Data are stratified into low and high altitude regions and summarized by the median (dots) and interquartile range (bars) of surveys available in each year. Smoothed lines show the LOWESS-smoothed moving average. *Pf*PR_2-10_: *Plasmodium falciparum* parasite rate in the age-standardized two-up-to-ten year old age range (Smith *et al*. 2007).

Summary time series plots for community parasite rate surveys in Kenya, Tanzania, Burundi, Rwanda, and Uganda are shown in Figure 5. In Kenya, the moving average lines for both low and high altitude regions display an overall decreasing trend across the period 1985-2008, although in low altitude regions this decline slows or reverses slightly during the period 1995-2000. In Tanzania, the high altitude trend line displays a similar overall decreasing pattern interrupted by a phase of increasing parasite rates in the middle years of the period. For low altitude areas an increasing trend is displayed up to 1996 but this is based on very few data points. After 1996 a clear decreasing trend is displayed. For the combined Burundi, Rwanda, and Uganda region, parasite rates in both high and low altitude regions are seen to generally increase until the late 1990s/early 2000s after which a marked decrease is displayed. Although based on an opportunistic assembly of available community parasite rate surveys, and despite substantial within- and between- year variation not explained by the smoothed trends, these simple summary plots point to a consistent and substantial decline in *P. falciparum* prevalence since 2002 or earlier across both low and high altitude regions of East Africa. These findings suggest that the recent decreases in case incidence described above for Kericho may be indicative of wider regional trends not limited to specific high-altitude sites. Such findings are also consistent with continental and global scale changes in *P. falciparum* endemicity described recently across much longer time periods [1].

## Discussion

We have re-examined temperature time series from locations in highland East Africa using the *t_DAN_* robust test for trends [11], [14]. Applying simple regression models evaluated using the *t_DAN_* robust t-test to the data from CRU that we previously used [4] finds positive but insignificant trends. However, the new CRU TS 2.1 and CRU TS 3.0 datasets shows a highly significant temperature increase in Kericho in both the 1966-1996 period examined by Chavez and Koenraadt [6], in the shorter 1970-1995 period examined by Hay *et al*. [4] and in the longer 1966-2006 period. The trend in Kericho is not, however, significant for the 1950-2002 period used by Pascual *et al* [8]. The trend is significant for East Africa as a whole at least the 5% level for both the TS 2.1 and TS 3.0 datasets for all the periods that we test.

Therefore, Chavez and Koenraadt's [6] general claim that, contrary to Hay *et al*. [4], temperature did increase in Kericho is correct but this is due to changes in the data used rather than any inadequacies of our statistical methods. The trend of increasing temperature has continued or accelerated further in the 1996-2006 period. These findings also explain the results of Pascual *et al*. [6] for the 1950-2002 period. But our results in this paper again show that there is no significant trend in the 1970-1995 period in the temperature data previously published by CRU. It is crucial when making claims for the empirical superiority of one statistical method over another to test both methods on the same datasets.

Our examination of the mean temperature series for Kericho prepared by Omumbo *et al*. [9] shows that again, adding post-1995 data results in a steeper trend. The 1979-1995 sample does not have a significant time trend. Hence, while high quality data is of value, use of the data from a single met station vs. use of the interpolated CRU database is not the reason that Hay et al. [4] failed to find a significant temperature trend for Kericho in the 1970 to 1995 period.

Finally, regardless of its etiology, malaria in Kericho and many other areas of East Africa has decreased during periods of unambiguous warming.
